# Presentation delay, misdiagnosis, inter-hospital transfer times and surgical outcomes in testicular torsion: analysis of statewide case series from central Brazil

**DOI:** 10.1590/S1677-5538.IBJU.2019.0660

**Published:** 2020-09-02

**Authors:** Aderivaldo Cabral Dias, Marcus Vinicius Osorio Maroccolo, Homero de Paula Ribeiro, Cassio Luis Zanettini Riccetto

**Affiliations:** 1 Unidade de Urologia Hospital de Base do Distrito Federal BrasíliaDF Brasil Unidade de Urologia do Hospital de Base do Distrito Federal, Brasília, DF, Brasil;; 2 Departamento de Cirurgia Faculdade de Ciências Médicas Universidade Estadual de Campinas CampinasSP Brasil Disciplina de Urologia, Departamento de Cirurgia, Faculdade de Ciências Médicas, Universidade Estadual de Campinas - UNICAMP, Campinas, SP, Brasil;; 3 Departamento de Cirurgia Hospital da Criança José de Alencar BrasíliaDF Brasil Departamento de Cirurgia, Hospital da Criança José de Alencar, Brasília, DF, Brasil

**Keywords:** Spermatic Cord Torsion, Diagnosis, Surgical Procedures, Operative

## Abstract

**Purpose:**

To estimate statewide presentation delay, misdiagnosis rate, inter-hospital transfer times and testicular salvage for testicular torsion patients treated in our state’s public health system.

**Patients and Methods:**

Case series of consecutive testicular torsion patients treated in our state’s public health system between 2012-2018. Predictors included presentation delay (time from symptoms to first medical assessment), facilitie’s level-of-care (primary, secondary, tertiary), first diagnosis (torsion, epididymitis, other), Doppler-enhanced ultrasound request (Doppler-US) and inter-hospital transfer times, with surgical organ salvage as the main response. We used Bayesian regression to estimate the effect of first examining facilitie’s level-of-care, first diagnosis, and Doppler-US on transfer time.

**Results:**

505 patients were included, most (298, 59%) with presentation delay >6 hours. Misdiagnosis at first examining facility raised transfer time from median 2.8 to 23.4 (epididymitis) and 37.9 hours (other) and lowered testicular salvage rates from 60.3% (torsion) to 10.7% (epididymitis) and 18.3% (other). Doppler-US had negligible effects on transfer time once controlling for misdiagnosis in the regression model. Although organ salvage in patients presenting before 6 hours at the tertiary facility was high (94.6%, and about 20% lower for those presenting at lower levels-of-care), the overall salvage rate was more modest (46%).

**Conclusion:**

Our low overall testicular salvage rates originated from a large proportion of late presentations combined with long transfer times caused by frequent misdiagnoses. Our results indicate that efforts to improve salvage rates should aim at enhancing population-wide disease awareness and continuously updating physicians working at primary and secondary levels-of-care about scrotal emergencies.

## INTRODUCTION

Intravaginal testicular torsion (henceforth testicular torsion) occurs when the testis rotates upon its axis inside the tunica vaginalis, twisting the spermatic cord or mesorchium ( 1 , 2 ) interrupting its blood supply. Left untreated, the almost universal outcome is testicular hemorrhagic infarction. The disease is a frequent cause of organ loss by either orchidectomy or atrophy, especially in emerging countries ( 3 , 4 ).

Since irreversible ischemic injury swiftly follows testicular torsion, supplemental imaging studies are not required after clinical diagnosis. One must admit, however, the possibility of diagnostic uncertainty, mainly acute epididymitis and torsion of testicular appendages ( 3 , 5 ). In these situations, imaging studies, usually Doppler-Enhanced Testicular Ultrasonography (Doppler-US), may be useful ( 6 , 7 ). In hierarchical health care systems such as the one we have in Brazil ( 8 ), where patients with scrotal emergencies are often first examined by general practitioners at primary or secondary level-of-care facilities, then referred to tertiary facilities for treatment, this not only requires the first examining physician to perceive the case as an emergency, but also that referral is not deferred for supplemental diagnostic testing. Thus, in the ideal situation - where patients rapidly seek medical attention, and are promptly examined by a competent diagnostician with immediate access to expert diagnostic imaging and referral protocols - one should observe low orchidectomy rates ( 9 ), which, unfortunately, have not been our experience ( 10 ).

While the time interval between symptoms and first medical assessment, i.e. presentation delay, rests upon the patient’s awareness of the disease and medical care availability ( 11 - 13 ), delays from this first assessment until evaluation at the treating institution have been associated with the first examining physician’s clinical diagnosis and subsequent action, such as imaging studies orders and inter-hospital patient referral and transfer ( 14 - 16 ). This information, however, comes from economically advanced countries, and although one may postulate that similar associations should also be observed in emerging countries, region-specific data is necessary to ascertain not only the existence but also the magnitude of these associations.

Since our tertiary unit is the referral center for testicular torsion patients within the public health network of our state - which grants database access for each patient’s entire medical history within the network, with time-stamps for each medical assessment - we may begin to fill this void in the urological literature. In this study, we set a two-fold goal. Firstly, to evaluate the relative roles of presentation delay and inter-hospital transfer time on testicular salvage; and secondly, to investigate the influence of the patient’s first clinical diagnosis and Doppler-US request on inter-hospital transfer time, especially on those examined shortly after symptoms, where expedient treatment is most decisive.

## PATIENTS AND METHODS

### Patient identification and variable recovery

After Institutional Board Review, all consecutive patients surgically diagnosed with testicular torsion in our tertiary facility between January 2012 and January 2018 were retrospectively identified from our unit’s data repository and were initially included in the study’s database. We retrieved each patient’s complete medical file - including assessments made outside our unit - for variable recovery. Since we did not have access to the time-stamps of patients first examined at private facilities or outside state borders, these were excluded from further analysis.

Continuous predictors included patient’s age in years, and the following time intervals: presentation delay, defined as the time between symptom’s onset and first examination, and transfer time, defined as the time between first examination and assessment at the tertiary facility. The arithmetic sum of these sub-intervals was named treatment delay. Categorical predictors included: The level-of-care of the facility where the patient was first examined (first examining facility), as defined by the Brazilian Health Authority ( 8 ): primary, secondary or tertiary; first diagnosis, defined as the clinical diagnosis recorded by the first examining physician: testicular torsion (torsion), acute epididymitis (epididymitis), neither torsion nor epididymitis (other); and whether Doppler-US was requested at the first examining facility, regardless of the actual performance of the study. Response variables were surgical outcome (whether or not the testicle was surgically salvaged) and transfer time, the latter with level-of-care, first diagnosis, and Doppler-US request as predictors.

### Univariate and bivariate analysis

Continuous variables were summarized by their medians and interquartile range (IQR), and categorical variables by their frequencies. Differences between continuous variables were assessed with Kruskal-Wallis´ (KW) and Dunn’s tests, and we evaluated differences between categorical variables with Pearson’s Chi-Square (Chi-Square) or Fisher’s Exact (Fisher) tests, with statistical significance set at <0.05.

### Linear regression

Bayesian hierarchical linear regression models were implemented using level-of-care, first diagnosis, and Doppler-US as predictors and transfer time as the response variable. The posterior distributions of the parameters were graphically presented and summarized by their medians and 0.95 Highest Posterior Density intervals (HPDI).

### Statistical software

Computations took place within the R language statistical environment ( 17 ), supplemented by the rjags ( 18 ) package.

### Reporting Guideline

In this report we followed the Preferred Reporting of CasE Series in Surgery (PROCESS) ( 19 , 20 ) guidelines.

## RESULTS

### Data recovery and first examining facility

**Figure 1:**
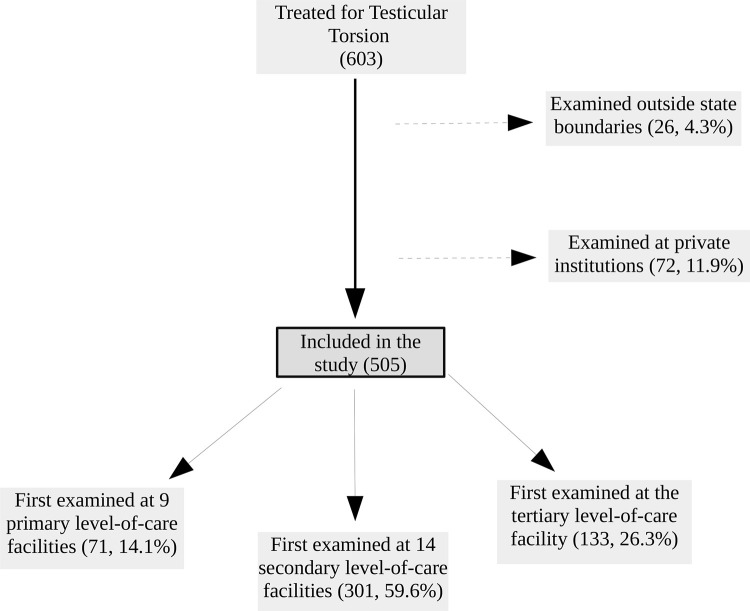
Distribution of patients according to the first examining facility level-of-care.

Six-hundred and three patients were treated for testicular torsion during the study period. We excluded 72 (11.9%) patients examined in private institutions and 26 (4.3%) patients examined outside state boundaries from our analysis. Most remaining 505 patients (median age 16.1 years, IQR 14.2-18.9) were first examined at secondary facilities (301, 59.6%; Figure-1 ). There were no missing data on recovered variables.

### Presentation delay, first examining facility and first diagnosis

**Figure 2:**
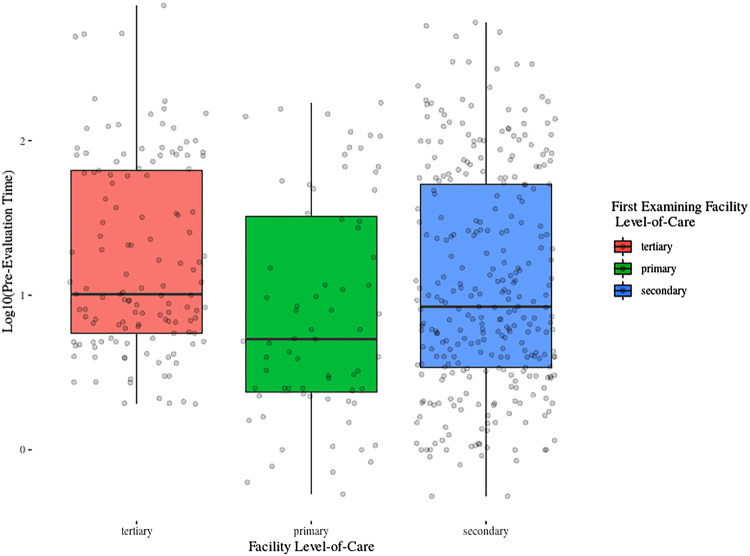
Presentation delay (log10 transformed) according to the first examining facilitie’s level-of-care.

Median presentation delay was 8.7 hours (IQR 3.9-53.4), with significant differences between primary (median 5.2 hours, IQR 2.4-32.3) and secondary facilities (median 8.4 hours, IQR 3.4-52.1; P=0.025), and between primary or secondary versus tertiary facilities (median 10.1 hours, IQR 5.7-64.2; P=0.004 and 0.008, respectively; Dunn’s; Figure-2 ).

Most patients were first diagnosed with torsion (345, 68.3%). Fifty-six (11.1%) were first diagnosed with epididymitis and 104 (20.6%) received another first diagnosis. Torsion was more often first diagnosed at the tertiary facility (123/133, 92.5%), compared to primary (44/71, 62%) and secondary facilities (178/301, 59.1%; P <0.001, Chi-Square; Table-1 ).

**Table 1:** Testicular Salvage by First Examining Facility, First Diagnosis and Doppler-US request.

First Examining Facility (N, %)	First Dx (N, %)	Doppler-US (N, %)	Salvage (%)
**Primary (71, 14.1%)**	Torsion (44/71, 62%)	Yes (5/44, 11.4%)	3/5 (60%)
		No (39/44, 88.6%)	18/39 (46.2% )
	Epidydimitis (7/71, 9.9%)	Yes (3/7, 42.9%)	2/3 (66.7%)
		No (4/7, 57.1%)	3/4 (75%)
	Other (20/71, 28.2%)	Yes (9/20, 45%)	3/9 (30%)
		No (11/20, 55%)	8/11 (72.7%)
**Secondary (301, 59.6%)**	Torsion (178/301, 59.1%)	Yes (40/178, 22.5%)	13/40 (24.1%)
		No (138/178, 77.5%)	54/138 (29%)
	Epidydimitis (47/301, 15.6%)	Yes (21/47, 44.7%)	10/21(47.6%)
		No (26/47, 55.3%)	11/26 (42.3%)
	Other (76/301, 25.2%)	Yes (42/76, 55.3%)	18/42 (42.9%)
		No (34/76, 44.7%)	15/34 (44.1%)
**Tertiary (133, 26.3%)**	Torsion (123/133, 92.5%)	Yes (4/123, 3.3%)	0
		No (119/123, 96.7%)	71/119 (59.7%)
	Epidydimitis (2/133, 1.5%)	Yes (0)	-
		No (2/2, 100%)	0
	Other (8/133, 6%)	Yes (2/8, 25%)	0
		No (6/8, 75%)	0

Legend to Table 1 - Patient distribution according to first examination facility level-of-care facility (Primary, Secondary, Tertiary), first diagnosis, Doppler-US request and testicular surgical salvage. First Dx, diagnosis at first examining facility, Torsion, testicular torsion; Epidydimitis, acute epidydimitis; Other, neither testicular torsion nor acute epidydimitis; Doppler-US, Doppler-enhanced testicular ultrasonography request at first examination; Salvage, surgical salvage of the testis; N, number of patients.

### Doppler-US at first examining facility by first diagnosis and presentation delay

Doppler-US was requested for 126 patients (25%), more often in primary (17/71, 23.9%) and secondary facilities (103/301, 34.2%) than in the tertiary facility (6/133, 4.5%; P <0.001, Chi-Square). Doppler-US was less frequently requested when the first diagnosis was torsion (49/345, 14.2%; compared to epididymitis: 24/56, 42.9%; or other: 53/104, 51%; P <0.001, Chi-Square).

Globally, Doppler-US requests were associated with longer presentation delays (median 24.3 hours, IQR 5.8-74.2 versus median 7.6 hours, IQR 3.6-33.8; P <0.001, KW), a pattern reproduced at primary (median 27.3 hours, IQR 6.0-62.7 versus median 3.6 hours, IQR 2.1-15.0; P=0.006) and secondary level-of-care facilities (median 22.8 hours, IQR 5.8-71.9 versus 6.0 hours, IQR 2.9-25.6; P <0.001, KW). Longer - but not statistically significant-presentation delays were also associated with Doppler-US requests at the tertiary level (median 65.9 hours, IQR 9.3-126.8, versus 10.1 hours, IQR 5.7-61.9; P=0.128, KW).

### Transfer time by first examining facility, first diagnosis, and Doppler-US

Transfer time was similar between primary and secondary facilities (median 5.7 hours, IQR 2.7-21.7 and 4.8 hours, IQR 2.1-22.5, respectively; P=0.276, KW). Transfer times diverged, however, considering first diagnosis: Median transfer time for patients diagnosed with torsion was 2.8 hours (IQR 1.8-5.7), compared to 37.9 hours (IQR 6.7-133.4; P <0.001, Dunn’s test) for epididymitis and 23.4 hours (IQR 5.9-69.7; P <0.001, Dunn’s test) for other diagnoses. Transfer time was longer when Doppler-US was requested, rising from median 3.5 hours (IQR 1.9-11.1), when not requested, to 10.5 hours (IQR 4.2-53.8), when requested (P <0.001, KW).

### Testicular salvage by first examining facility, first diagnosis, and Doppler-US request

The testis was surgically salvaged in 233 patients (46.1%). Although salvage rates were similar across levels-of-care (primary: 30/71, 42.3%; secondary: 132/301, 43.9%; tertiary: 71/133, 53.4%; P=0.152, Chi-Square), rates differed regarding patient’s subsets presenting before 6 and 3 hours: In the former time frame, 35/37 (94.6%) patients first examined at the tertiary facility had their organs salvaged, compared to 24/39 (61.5%) first examined at primary and 100/131 (76.3%) first examined at secondary facilities (P=0.002, Chi-Square). Similar differences were observed in the subset of patients with presentation delay <3 hours, where all 11 patients first examined at the tertiary facility had their organs salvaged, contrasting with 18/25 (72%) and 50/65 (76.5%) in those first seen at primary and secondary facilities (P <0.001, Chi-Square).

Most patients first diagnosed with torsion had their organs salvaged (208/345, 60.3%), in contrast with those first diagnosed with epididymitis (6/56, 10.7%) or other (19/104, 18.3%; P <0.001, Chi-Square). Salvage rates for 160 patients first diagnosed with torsion and presentation delay <6 hours were 73.3%, 90.3% and 94.3% for those respectively seen at primary, secondary and tertiary facilities. In the subset of patients first diagnosed with testicular torsion with presentation delay <3 hours, Doppler-US was associated with significantly lower salvage rates: 28/126 (22.2%) organs were salvaged when Doppler-US was requested, compared to 205/378 (54.2%, P <0.001, Chi-Square) when it was not requested.

### Bayesian regression of transfer time by Doppler-US and first diagnosis

**Figure 3:**
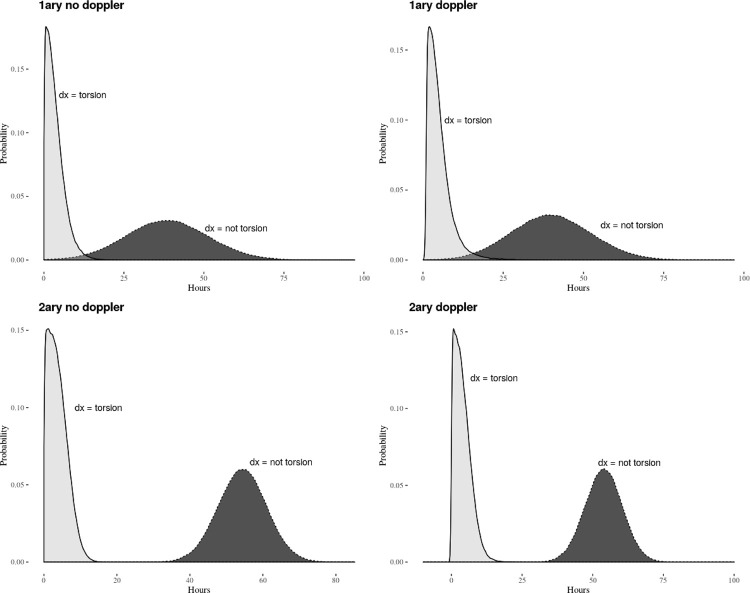
Distribution of transfer times according to facilities´ level of care (1ary=primary, 2ary=secondary), first diagnosis (dx=torsion, dx=not torsion) and Doppler-US request computed from the Bayesian linear regression model. Horizontal axis, transfer time in hours; vertical axis, probability.

Models were implemented for the whole dataset and patient’s subsets with presentation delay <6 and <3 hours, with 300.000 to 400.000 posterior distribution samples generated for each model. Testicular torsion misdiagnosis increased overall median transfer times by 53.2 hours (HPDI 46.1-60.3, Table-2 , Figure-3 ), an effect also observed in the <6 hours (median increase 43.5 hours) and in the <3 hours (median increase 40.2 hours) subsets. Doppler-US requests had negligible effects on transfer time once controlling for misdiagnosis.

**Table 2:** Bayesian linear regression model output.

Level-of-Care	Dx at First Examining Facility	Tranfer Time (median, HPDI)		
		All patients	PD <6 h	PD <3 h
		N = 372	N = 207	N = 101
**Primary**				
	Testicular torsion, Doppler-US	6.5 (0.1 – 15.7)	2.6 (0.1 – 10.9)	2.8 (0.1 – 9.5)
	Testicular torsion, no Doppler-US	6.5 (0.1 – 14.9)	2.5 (0.1 – 8.94)	2.6 (0.1 – 7.8)
	Other Dx, Doppler-US	52.4 (26.6 – 77.9)	38.6 (13.5 – 63.7)	32.6 (10.7 – 54.3)
	Other Dx, no Doppler-US	52.6 (27.6 – 78.8)	38.4 (13.2 – 63.5)	32.6 (10.9 – 54.4)
**Secondary**				
	Testicular torsion, Doppler-US	8.8 (0.3 – 17.5)	2.8 (0.1 – 9.4)	3.6 (0.1 – 9.5)
	Testicular torsion, no Doppler-US	8.6 (0.1 – 16.8)	3.2 (0.1 – 8.6)	3.4 (0.1 – 8.5)
	Other Dx, Doppler-US	69.1 (56.5 – 81.6)	53.9 (40.9 – 66.8)	53.3 (40.0 – 66.0)
	Other Dx, no Doppler-US	68.9 (56.4 – 81.4)	54.4 (41.2 – 67.3)	53.4 (40.4 – 66.3)

Legend for table 2 - Bayesian linear regression model output. Level-of-care, medical facility’s level of care (primary, secondary); Dx at First Examining Facility, diagnosis at the first examining facility; Transfer Time, time-interval in hours between first examination and examination at the tertiary facility, estimated for all patients as well as for patients with presentation delay (PD) <6 and <3 hours; Doppler-US, request for testicular Doppler-enhanced ultrasonography; HPDI, 0.95 highest posterior density interval.

## DISCUSSION

In this study, clinical misdiagnosis of testicular torsion at first examination, at either primary or secondary level-of-care facilities, was associated with large increases in inter-hospital transfer time. These long transfer times combined with even longer presentation delays to yield lowed an overall testicular salvage rate (46.1%). The highest salvage rates were observed among patients first examined at the tertiary facility shortly after symptoms.

Most of our patients (59%) were first examined after 6 hours of symptom’s onset. Presentation delay has been reported as the leading cause of organ loss in testicular torsion ( 21 ), having been associated with low patient and parental awareness both of the disease and of its limited time frame for treatment. British researchers ( 11 ) found that only 30% of their patients sought medical attention within 6 hours of symptoms (46% presented after 12 hours), and 2/3 of those were incognizant that the disease could cause organ loss. Similarly, American investigators ( 12 ) reported that only 1/3 of 479 parents surveyed in a Pediatric Urology clinic had some knowledge about the disease, and an Irish study ( 13 ) uncovered that although 56% of parents knew about the disease, just 1/3 were aware of the time frame available for treatment.

Even if the patient rapidly seeks medical attention, the organ can still be lost if the first examining physician does not contemplate the diagnosis of testicular torsion. In our series, misdiagnosis was second only to presentation delay in determining the organ’s prognosis-especially in patients presenting before 6 hours of symptoms-by increasing median transfer time by more than a day on average. This influence of patient’s first diagnosis on transfer time remained even after accounting for Doppler-US in the regression model. Since the request for supplemental imaging studies must chronologically follow the patient’s first examination and initial diagnosis, this finding suggests that Doppler-US mainly acted as a mediator of the effects of first diagnosis on the outcome.

Difficulties with the differential diagnosis of acute testicular disease could be addressed by the dissemination of a predictive tool such as the Testicular Workup for Ischemia and Suspected Torsion (TWIST) score ( 22 , 23 ), which attributes points to medical history and physical examination findings (testicular swelling=2, hardened testicle=2, cremasteric reflex absent=1, nausea/vomiting=1 and high-riding testis=1), with scores ≤2 representing low risk, 3-4 intermediate and >4 high risk for testicular torsion. Patients with high scores should be promptly operated, those with low scores should be followed, and further investigation would only be warranted for patients with intermediate scores ( 24 ). Furthermore, the score has high positive and negative predictive values even when used by non-physicians ( 25 ).

Inter-hospital transfer may further delay treatment, with repercussions on salvage rates. In our study, even patients with presentation delay <3 hours and correctly diagnosed at primary or secondary facilities had salvage rates 10% lower than those first presenting at the tertiary facility. These findings agree with Bayne’s ( 26 ), that reported a positive association between inter-hospital transfer and orchidectomy, especially in boys with presentation delay <24 hours. Likewise, Preece and associates ( 14 ) found that patients transferred within 24 hours of symptoms to their tertiary hospital had twice the probability of testicular loss (30%, versus 15% for those first seen at their tertiary hospital). Although investigators from California ( 15 ) described comparable salvage rates between patients transferred or examined at their tertiary hospital, the mean time from symptoms to operating room between them differed by only 83 minutes.

Our results show that there is much to improve in the care of our testicular torsion patients. Despite addressing the main cause of organ loss-long presentation delay-attempts to instruct the public about testicular torsion can be complex and expensive to implement, likely requiring large-scale information campaigns. Alternatively, educational efforts aimed at the medical community to increase knowledge about the disease and to improve its diagnosis could be less costly and produce faster results, as envisioned by Friedman and associates ( 27 ). These authors developed a Computer-Enhanced Visual Learning tool (accessible from the CEVL tab at www.jpurol.com) that reviews genital male anatomy and differential diagnosis of scrotal emergencies, supplemented with a testicular torsion likelihood calculator that uses as inputs easily obtainable clinical data (but not the TWIST score).

In our series, only patients that presented at the tertiary facility shortly after symptoms had salvage rates >90%, which we attribute to our experience with this disease. Yet, we could not help wondering about the possibility of reproducing these results at the secondary level-of-care, as testicular torsion’s diagnosis is eminently clinical and its surgical treatment is not complex. Matter-of-fact, it was often performed by general surgeons before the consolidation of urologic specialty ( 28 ). Patients could also benefit from manual detorsion at the emergency setting, a time-tested maneuver ( 29 , 30 ) within reach of any physician. Already in the 1960s, Dr. Sparks, a medical officer working in the small English town of Rugby, demonstrated that this disease could be successfully treated at a lower level-of-care ( 31 ). Dr. Sparks combined diagnostic shrewdness, ample use of manual detorsion and timely surgical exploration to salvage all but one testicle of his 15 testicular torsion patients-a commendable 93.4% salvage rate.

This study has many limitations. The main one, shared by all retrospective studies, is selection bias, since our database contained only surgically treated testicular torsion patients and did not provide means to identify those clinically treated for other acute scrotal diseases. Selection bias was manifested in the overall low proportion of Doppler-US requests, especially at the tertiary facility, where patients were first assessed by a team attentive to the recommendation to waive supplemental studies after testicular torsion was clinically diagnosed. Recall bias regarding presentation delay should also be considered, since information about symptom’s onset is patient/parent-provided, with inherent uncertainty that increases with time. Lastly, since our data comes from the smallest Brazilian state with the highest per capita income, further endowed with a structured public health network, we advise caution in generalizing our results.

## CONCLUSIONS

In this large statewide case series, we observed that most testicular torsion patients first presented, after considerable delay, at primary or secondary level-of-care facilities, where they were often clinically misdiagnosed. Misdiagnosis led to the increase in inter-hospital transfer time, and the cumulative effect of these extended presentation delays and transfer times led to a low overall organ salvage rate.

This study provides relevant and previously unavailable contemporary information to apprise our medical - especially urological - communities to the real-world state-of-affairs of testicular torsion assessment and treatment at our public health care system. We hope that this study motivates other investigators to replicate our initiative, in order to provide a wider view of the current situation of testicular torsion care and determine where we should act to improve testicular torsion outcomes.

## ABBREVIATIONS

Doppler-US: Doppler-enhanced ultrasonography
IQR: Interquartile ratio
HPDI: Highest posterior density interval
PROCESS: Preferred Reporting of CasE Series in Surgery
